# Optimal Sequential Diagnostic Strategy Generation Considering Test Placement Cost for Multimode Systems

**DOI:** 10.3390/s151025592

**Published:** 2015-10-08

**Authors:** Shigang Zhang, Lijun Song, Wei Zhang, Zheng Hu, Yongmin Yang

**Affiliations:** 1 Laboratory of Science and Technology on Integrated Logistics Support, College of Mechatronics Engineering and Automation, National University of Defense Technology, Changsha 410073, China; E-Mails: zhenghu@nudt.edu.cn (Z.H.); yangyongmin@163.com (Y.Y.); 2 State key Laboratory of Robotics, Shenyang Institute of Automation, Chinese Academy of Sciences, Shenyang 110016, China; E-Mail: zhangwei@sia.cn; 3 College of Basic Education, National University of Defense Technology, Changsha 410073, China; E-Mail: junlisong@nudt.edu.cn

**Keywords:** diagnostic strategy, sequential fault diagnosis, AND/OR graph, multimode system

## Abstract

Sequential fault diagnosis is an approach that realizes fault isolation by executing the optimal test step by step. The strategy used, *i.e*., the sequential diagnostic strategy, has great influence on diagnostic accuracy and cost. Optimal sequential diagnostic strategy generation is an important step in the process of diagnosis system construction, which has been studied extensively in the literature. However, previous algorithms either are designed for single mode systems or do not consider test placement cost. They are not suitable to solve the sequential diagnostic strategy generation problem considering test placement cost for multimode systems. Therefore, this problem is studied in this paper. A formulation is presented. Two algorithms are proposed, one of which is realized by system transformation and the other is newly designed. Extensive simulations are carried out to test the effectiveness of the algorithms. A real-world system is also presented. All the results show that both of them have the ability to solve the diagnostic strategy generation problem, and they have different characteristics.

## 1. Introduction

Diagnostic strategy generation is one of the important contents of diagnosis system design. It has great influence on diagnostic accuracy and cost. As an efficient diagnostic method, sequential diagnostic strategy, which realizes fault isolation by executing the optimal test step by step, has been applied extensively, and thus extensive attention has been paid to how to generate it optimally. The problem is also called test sequencing problem [1] or sequential fault diagnosis problem [2]. One of the typical methods is to formulate it as an AND/OR graph search problem and solve it using an AO* algorithm [3], where a good result has been obtained for the perfect test systems. Based on the results, in order to solve the problem in real-world systems where unreliable tests and multiple faults may exist, heuristic functions (information gain heuristic, rollout strategy, *etc.*) have been developed by different researchers [4,5,6]. These methods achieve good effect in generating of optimal diagnostic strategy. However, only the execution cost of the tests (power consumption, time to carry out the test, *etc*.) is considered in the traditional methods, and the diagnostic strategy obtained is not optimal from the viewpoint of life cycle [7]. Specifically, the sensors and diagnostic steps are optimized based on the object that the average shortest diagnostic path is obtained, which indicates that the sensor placement cost (purchase cost, installation cost, *etc.*) is omitted in the algorithm and thus the result is actually not optimal as expected. Consequently, an algorithm (the AOL algorithm) was proposed in our previous research to solve the problem [7]. Based on the result, the algorithm is further developed to solve the problem with imperfect tests by generating sub-tree using information heuristics [8]. Both of the methods in [7,8] can generate a better result than the previous algorithms from the viewpoint of life cycle cost.

However, these two methods were designed for single mode systems, *i.e*., the dependency relationships between the faults and the tests are assumed to be changeless whichever work station the equipment is. This assumption may be true for a simple system, but it is not feasible for complicated systems, which are very common in the real world.

Take a satellite as an example—it can work using either solar panels or batteries. The solar panel can also be divided into different individual parts. If we encounter a power supply problem in this spacecraft, different suspect failure sources will be obtained in the two operation modes which contain either solar panels or batteries. This means that the D-matrix (diagnostic dictionary matrix) is different in this kind of multimode systems and will undoubtedly affect the diagnostic strategy. This situation is very common in redundant systems and systems with different working status. Mode change should be taken into account to generate the optimal diagnostic strategy. For this problem, Ruan *et al.* proposed an algorithm based on information gain heuristics [9]. Rollout strategy is applied to improve the result. Yang *et al*. proposed a quasi multi-step look-ahead search algorithm, which can balance between diagnostic accuracy and computational complexity [10]. However, as discussed above, their methods do not consider the test placement cost, which means that their optimality is not as good as expected. Further research must be carried out to solve this problem.

In this paper, the optimal sequential diagnostic strategy generation problem considering test placement cost for multimode systems is studied. It is formulated as an AND/OR graph search problem. Two solution algorithms are proposed. Computational experiments are carried out to compare and test the effectiveness of the methods. A real-world system is also presented.

The remainder of the paper is organized as follows: in Section 2, the problem studied in this paper is formulated. In Section 3, two algorithms are proposed, one of which is realized by system transformation and the other is newly designed. In Section 4, the proposed algorithm is extended to deal with the problem with imperfect tests. In Section 5, the model and algorithms are tested and compared on various simulated systems. In Section 6, the proposed algorithms are tested on a real-world system. Finally, the paper concludes with a summary in Section 7.

## 2. Problem Formulation

In the diagnostic strategy generation problem for the multimode systems, not only the test costs should be considered, but also the mode transition costs should be taken into account. Formally, this problem consists of the following:

(1) A system consisting of k + 1 fault states, $S=\{s_{1},\cdots ,s_{k},s_{k+1}\}$, where $s_{k+1}$ denotes the fault-free state.
(2) The failure rate corresponding to each fault state in S, $P=\{p_{1},\cdots ,p_{k},p_{k+1}\},$ and they are normalized, *i.e*., $\sum_{i=1}^{k+1}p_{i}=1$.
(3) A finite set of candidate tests $T=\{t_{1},t_{2},\cdots ,t_{n}\}$.
(4) Test placement cost $CP=\{CP_{1},CP_{2},\cdots ,CP_{n}\}$ and execution cost $CE=\{CE_{1},CE_{2},\cdots ,CE_{n}\}$ corresponding to each test in T.
(5) A finite set of L system modes denoted by $M=\{m_{1},m_{2},\cdots ,m_{L}\}$ and their transition cost $C={\left[c_{ij}\right]}_{L\times L}$, where $c_{ij}$ denotes the cost occurred when the mode is changed from $m_{i}$ to $m_{j}$. It is evident that the main diagonal elements are zero, *i.e*., $c_{ij}=0$ for i=j.
(6) A series of diagnostic dictionary matrixes (D-matrix) $D_{l}={\left[d_{ij}^{l}\right]}_{(k+1)\times n},$ l=1,⋯,L, where $d_{ij}^{l}$ is 1 if test $t_{j}$ can detect fault $s_{i}$ at mode l, and 0 otherwise. For the fault-free state, $d_{(k+1)j}^{l}=0,j=1,\cdots ,n,\;l=1,\cdots ,L$. Note that we assume all the tests are available at each mode, which will simplify the derivation process. In case that a test cannot be used in a mode, only needs to set all the elements in the D-matrix corresponding to the test at the mode to zero.
(7) Execution times N of the sequential fault diagnosis strategy in the life cycle period, which can either be obtained from the historical data or be calculated from the reliability data [7].

**Figure 1:**
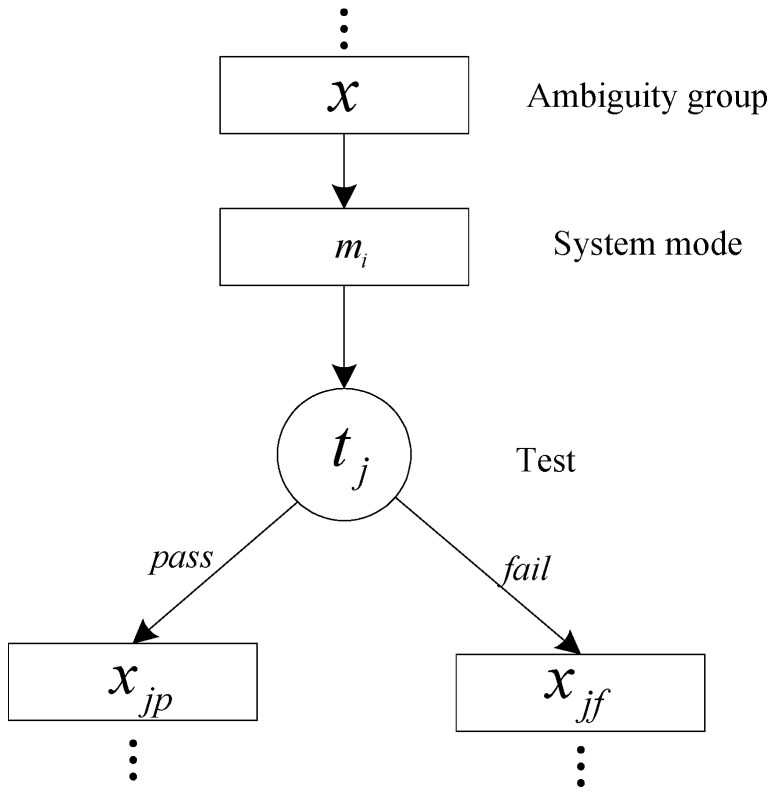
A typical diagnostic strategy of multimode system.

Diagnostic strategy generation problem is an optimization problem. Its objective is to obtain a diagnostic tree achieving the maximum diagnostic accuracy with the minimum test cost. It is usually formulated as a binary AND/OR graph search problem in the single-mode system [7], *i.e.*,

(1) $$\min J=N\cdot J_{e}+J_{p}$$

where J denotes the total cost of the diagnostic strategy, $J_{p}$ denotes the test placement cost at the design stage and $J_{e}$ denote the average test execution cost of the diagnostic tree. Unlike the single-mode system, the mode transformation cost and dependency change should be considered when a system has more than one mode. The typical structure of a multimode system diagnostic strategy is shown in Figure 1.

Formally, $J_{e}$ in Equation (1) can be given by [9]:

(2) $$J_{e}=\sum_{i=1}^{k+1}\left\{\sum_{j=1}^{\left|\rho_{i}\right|}CE_{\rho_{i}[j]}+\sum_{j=1}^{\left|\rho_{i}\right|-1}c_{Q_{i}(j)Q_{i}(j+1)}\right\}p_{i}$$

where $\rho_{i}$ denotes the sequence of tests applied to isolate the fault state $s_{i}$ and $\left|\rho_{i}\right|$ denotes the cardinality of the test sequence $\rho_{i}$, $Q_{i}(j)$ denotes the mode index of the *j*th test in $\rho_{i}$.

$J_{p}$ is the summation of the placement cost of all the tests applied:

(3) $$J_{P}=\sum_{x\in \cup_{i=1}^{k+1}\rho_{i}}CP_{x}$$

Our problem is to design an algorithm to generate a diagnostic strategy that realizes Equation (1). This problem is NP-hard, which means that a useful optimal algorithm can hardly be found, and a feasible suboptimal algorithm is what we want to develop. Actually, it has been proved that the construction of optimal test sequence in the single-mode system is a NP-complete problem [11,12,13]. For a system with m tests and each of the test has n results, at most $n^{m‒2}m!$ diagnostic strategies can be obtained even when the test placement cost is not taken into account [14].

## 3. Solution Algorithms

### 3.1. Algorithm 1

As discussed above, previous algorithms cannot solve the diagnostic strategy generation problem considering test placement cost for multimode systems. In this section, we propose a method by means of system transformation. For the system formulated in Section 2, we generate a single mode systemas follows:

(4) $$T=\{t_{1},t_{2},\cdots ,t_{n^{\prime }}\}$$

where:

(5) $$n^{\prime }=L\cdot n$$

For the test placement cost:

(6) $$CP^{\prime }=\{CP_{1},CP_{2},\cdots ,CP_{n},CP_{n+1},\cdots ,CP_{n^{\prime }}\}$$

where $CP_{((i-1)\mathrm{mod}\;n)+1}=CP_{i}$.

For the test execution cost:

(7) $$CE^{\prime }=\{CE_{1},CE_{2},\cdots ,CE_{n},CE_{n+1},\cdots ,CE_{n^{\prime }}\}$$

where $CE_{((i-1)\mathrm{mod}\;n)+1}=CE_{i}$

The total mode number becomes $L^{\prime }=1$. The size of D-matrix becomes $(k+1)\times n^{\prime }$, *i.e*.,

(8) $$D={\left[{\hat{d}}_{ij}\right]}_{(k+1)\times n^{\prime }}$$

where:

(9) $${\hat{d}}_{ij}=d_{i(((j-1)\mathrm{mod}\;n)+1)}^{\left\lfloor \frac{j-1}{n}\right\rfloor +1}$$

Here, ⌊x⌋ denotes the floor function, *i.e*., the largest integer not greater than x. The other parameters are the same as those formulated in Section 2.

From the transformation process, it is evident that the dependency relationships between the tests and the faults are not changed. All of them are presented in a single mode system by extending the columns of the D-matrix. Then, the problem can be solved using the algorithm for single mode system. The drawback is that the mode transformation cost should be omitted in the algorithm, which may affect the effectiveness of the algorithm. Therefore, we propose a new algorithm in the next section.

### 3.2. Algorithm 2

According to the structure of the diagnostic strategy for the multimode system, the solution algorithm involves test selection and mode selection at each step. On the condition that the system mode is determined, the best test for ambiguity group x should be selected using the same strategy as the one in single mode system, that is [7]:

(10) $$j^{*}=\arg\underset{j}{\min}\{N\cdot \hat{P}(x)\cdot [CE_{j}+P(x_{jp})J_{e}(x_{jp})+P(x_{jf})J_{e}(x_{jf})]+J_{p}(x,j)\}$$

where $x_{jp}$ and $x_{jf}$ denote the pass and fail nodes respectively. $P(x_{jp})$ and $P(x_{jf})$ denote the probabilities of the test outcomes, where $P(x_{jp})+P(x_{jf})=1$. $J_{e}(x_{jp})$ and $J_{e}(x_{jf})$ are the cost-to-go of execution cost from $x_{jp}$ and $x_{jf}$, *i.e*., the cost in each branch. $J_{p}(x,j)$ is the test placement cost of the diagnosis strategy from x if $t_{j}$ is selected as the next test of node x. $\hat{P}(x)$ is the probability of state x in the diagnostic strategy.

Then, how to select the proper mode becomes the crux of the matter. A parameter denoting the ability of each mode needs to be designed. Here, let k denote current mode, the ability of mode $m_{i}$ is formulated as:

(11) $$A(m_{i},x)=\frac{H(m_{i},x)}{C(m_{i},x)}=\frac{-\sum_{j=1}^{p}p(l_{h_{j}})\log p(l_{h_{j}})}{c_{ki}+\sum_{j=1}^{p}p(l_{h_{j}})c(x,l_{h_{j}})}$$

where $LS=\{l_{h_{1}},l_{h_{2}},\cdots ,l_{h_{p}}\}$ denotes the leaf nodes of the sub-tree generated from x using information gain heuristic method, $p(l_{h_{j}})$ is the relative probability of the leaf node $l_{h_{j}}$, and:

(12) $$p(l_{h_{j}})=\frac{\sum_{s_{i}\in l_{h_{j}}}p_{i}}{\sum_{s_{i}\in x}p_{i}}$$

In Equation (11), $c(x,l_{s_{i}})$ denotes the test cost in the branch from x to $l_{s_{i}}$, $c_{ki}$ is the transformation cost from mode k to i. Actually, $H(m_{i},x)$ can be explained as the fault isolation ability of mode $m_{i}$, and $C(m_{i},x)$ denotes the estimated cost in mode i to achieve the expected fault isolation level.

After the formulation to select the best mode and the best test at each step is determined, the algorithm to generate diagnostic strategy considering test placement cost for multimode systems is designed based on the following idea and steps:

**Step 1** Initialize the diagnostic strategy with the root node.
**Step 2** Iterate the following steps until the satisfied diagnostic strategy is obtained.
  **Step 2.1** Determine the node to be expanded.
  **Step 2.2** Select the next best system mode.
  **Step 2.3** Expand the selected node for one step.
  **Step 2.4** Prepare for the cost-revising and arc-marking stages process.
  **Step 2.5** Iterate the following steps until all the revisions are carried out.
    **Step 2.5.1** Select the bottom node as the one to be revised.
    **Step 2.5.2** Recalculate the next best test of the selected node, and update its parameters.
    **Step 2.5.3** Determine upward nodes that may need cost revision, continue the revision process.

The algorithm mainly include expansion process and cost/branch revision process, which is similar to the structure of AO* algorithm. Details are shown as follows:

**Step 1**  Initialize a graphG consisting of the root node $x_{r}=\{p_{1},p_{2},\cdots ,p_{k+1}\}$. Label the root node as unsolved, and assume that the initial mode of the system is $m_{1}$.
**Step 2**  Repeat the following steps until the root node $x_{r}$ is labeled solved. Exit with $J=N\cdot J_{e}(x_{r})+J_{p}(x_{r})$ as the total cost and the marked tree as the fault diagnostic strategy.
  **Step 2.1**  Compute a partial graph $G^{\prime }$ by tracing down the marked arcs from the root node $x_{r}$. Select a leaf node $x^{\prime }$ in $G^{\prime }$ that maximizes H(x) as the node to be expanded, where x denotes a leaf node of $G^{\prime }$. H(x) denotes the entropy of the x and $H(x)=-\sum_{\pi_{i}\in x}\pi_{i}\log(\pi_{i})$, where $\pi_{i}$ is the probability of fault $s_{i}$ in x. Let $m_{l^{\prime }}$ denote system mode of the leaf $x^{\prime }$.
  **Step 2.2**  Calculate the diagnostic capability of each system mode from $x^{\prime }$via Equation (11), select the mode $m_{i^{\prime }}$ by $i^{\prime }=\arg\underset{1\leq i\leq L}{\max}A(m_{i},x^{\prime })$, and set it as the system mode in this branch.
  **Step 2.3**  let $T_{ui^{\prime }}(x^{\prime })$ denote the tests that have been used on the path from the root node to $x^{\prime }$ in mode $m_{i^{\prime }}$. For all the tests $t_{j}\notin T_{ui^{\prime }}(x^{\prime })$ in mode $m_{i^{\prime }}$, generate the successive nodes denoted by ${x^{\prime }}_{jp}$ and ${x^{\prime }}_{jf}$ corresponding to the pass and fail outcomes. Without changing the system mode, generate the sub-tree from ${x^{\prime }}_{jp}$ and ${x^{\prime }}_{jf}$ using information heuristic until the stopping criterion is satisfied. Let $T_{r}({x^{\prime }}_{jp}),\;T_{r}({x^{\prime }}_{jf})$ denote the tests applied in the sub-tree from ${x^{\prime }}_{jp}$ and ${x^{\prime }}_{jf}$ respectively. Calculate the execution costs of the sub-trees using the similar equation shown in Equation (2), and denote them as $J_{e}({x^{\prime }}_{jp})$ and $J_{e}({x^{\prime }}_{jf})$. Set $T_{ui^{\prime }}({x^{\prime }}_{jp})=T_{ui^{\prime }}({x^{\prime }}_{jf})=T_{ui^{\prime }}(x^{\prime })\cup \{t_{j}\}$, $\hat{P}({x^{\prime }}_{jp})=\hat{P}(x^{\prime })P({x^{\prime }}_{jp})$, $\hat{P}({x^{\prime }}_{jf})=\hat{P}(x^{\prime })P({x^{\prime }}_{jf})$. If either ${x^{\prime }}_{jp}$ or ${x^{\prime }}_{jf}$ is a terminal leaf node (satisfying the specified stopping criterion), label it as solved.
  **Step 2.4**  Define a set Z to denote the nodes in the graph G, and initialize it as $Z=\{x^{\prime }\}$.
  **Step 2.5**  Repeat the following steps until Z=∅
    **Step 2.5.1**  Remove from Z a node y such that no successor of y in G occurs in Z. Let $T_{r}^{\prime }(y)=T_{r}(y)$, $J_{e}^{\prime }(y)=J_{e}(y)$ and $J^{\prime }(y)=J(y)$
    **Step 2.5.2**  For *y*, calculate $J^{*}$ via Equation (10). Update $J_{e}(y)$ via Equation (13). Set $T_{r}(y)=T_{r}(y_{j^{*}p})\cup T_{r}(y_{j^{*}f})\cup \{t_{j^{*}}\}$, and $J(y)=J(y,j^{*})$. If both $y_{j^{*}p}$ and $y_{j^{*}f}$ are labeled as solved, label y as solved. Mark the arcs $y\to t_{j^{*}}$, $t_{j^{*}}\to y_{j^{*}p}$ and $t_{j^{*}}\to y_{j^{*}f}$.
    **Step 2.5.3**  if y is the root node, or $J_{e}^{\prime }(y)=J_{e}(y)$ and $T_{r}^{\prime }(y)=T_{r}(y)$ are satisfied simultaneously, continue to Step 2.4.1. Otherwise, add to Z all the ancestors of y along the marked arcs. Ignore the mode transition nodes.

In the algorithm:

(13) $$J_{e}(y)=CE_{j^{*}}+P(y_{j^{*}p})J_{e}(y_{j^{*}p})+P(y_{j^{*}f})J_{e}(y_{j^{*}f})$$

As the tests are perfect:

(14) $$\hat{P}(x)=\sum_{s_{i}\in x}p_{i}$$

(15) $$P(x_{jp})=\frac{\sum_{s_{i}\in x_{jp}}p_{i}}{\hat{P}(x)}$$

For the root node, it is evident that:

(16) $$\hat{P}(x_{r})=1$$

Note that is if the initial mode of the system is not $m_{1}$, one only needs to change the order of the system modes. The algorithm will not change. The stopping criterion is either the fault has been isolated or none of the unused test has the ability to distinguish the faults in the ambiguity group. Information heuristics to generate the sub-tree can be found in [4]. In order to improve the computation efficiency of the algorithm for large-scale system, two strategies are optional:

(1) In Step 2.3, not all the tests $t_{j}\notin T_{ui^{\prime }}(x^{\prime })$ in mode $m_{i^{\prime }}$ need to be selected as the next possible test node and generate a sub-tree. It is recommended that only a number of tests with the best information gain are considered and recorded, which will not affect the result obviously.
(2) The maximum backtrack steps can be set to reduce the computational time, which is very useful for the cases with imperfect tests, as discussed in the next section.

## 4. The Imperfect Cases

The problem formulated above is based on perfect test assumption, *i.e.*, the element in the D-matrix $d_{ij}^{l}$ either equals 1 or 0, which means a test detects a fault with a probability either of 100% or of 0. This may be not true in the real world systems because of electromagnetic interference, unreliable sensors, environmental conditions and so on. Actually, test sequencing problem based on imperfect data has been studied extensively in the literature [5,15,16]. The algorithm proposed in this paper can also be used to solve imperfect test problems by means of several modifications.

Specifically, the D-matrix firstly needs revision to represent imperfect tests, *i.e*., $D_{l}={\left[d_{ij}^{l}\right]}_{(k+1)\times n},$ l=1,⋯,L where $0\leq d_{ij}^{l}\leq 1$ denotes the detection probability of test $t_{j}$ to faul $s_{i}$ at mode l, *i.e*.:

(17) $$d_{ij}^{l}=\text{Prob\{test }t_{j}\text{fails at mode }l\text{ |}s_{i}\text{ occurrs\}}$$

The structure of the algorithm needs not to be changed. The difference is the information heuristic to generate the sub-tree and the failure probability after a test is applied. The algorithm can be found in [4].

## 5. Simulation Experiment

In this section, the algorithms proposed in this paper are tested extensively on simulated systems. Simulations were carried out in MATLAB, on a PC with 2.4 GHz CPU, 8 GB RAM. They are divided into perfect test scenario and imperfect test scenario. The results are averaged over 100 Monte Carlo runs. In all the simulations, the same stopping criterion is employed, which means that the diagnostic strategies generated by the two algorithms have the same diagnostic accuracy for the same case. The cost of the strategy is our major concern.

*● Perfect Test Scenario*

In this scenario, all the tests are assumed to be perfect, *i.e*., $d_{ij}^{l}$ in the D-matrix either equals 1 or 0, and it is generated randomly. Two kinds of systems of different scale are simulated. The following metrics and notation are used to evaluate the performance of our algorithms:

(1) m: number of failure modes in the system.
(2) n: number of tests in the system.
(2) *N*: execution times of the sequential fault diagnosis strategy, which denotes the ratio between the test placement cost and the test execution cost and it is selected in {10,100,1000} in the simulation.
(4) *L*: number of system modes.
(5) *mc*: maximum mode transition cost, which indicates that the mode transition cost is generated randomly in [0, *mc*]. Here, *mc* can be interpreted as the average proportion of the mode transition cost and the test cost.
(6) Algorithm 1: the algorithm presented in Section 3.1.
(7) Algorithm 2: the algorithm proposed in Section 3.2.
(8) Time: average computational time of the corresponding algorithm.
(9) Cost: total cost of the generated diagnostic strategy.
(10) *ratio*: proportion between the total cost of the diagnostic strategy generated by Algorithm 1 and by Algorithm 2, *i.e*.,
  (18) $$ratio=\frac{\text{Cost of Algorithm 1}}{\text{Cost of Algorithm 2}}$$

In the simulations, the fault probability, test execution cost and placement cost are generated randomly in [0,1]. The results in different scenarios are shown in Table 1, Table 2, Table 3 and Table 4.

**Table 1:** Simulation result for the perfect test cases (N = 100, L = 3).

System Scale	*mc*	Algorithm 1		Algorithm 2		*Ratio*
		Time(s)	Cost	Time(s)	Cost	
m = 10,n = 15	1	0.078	167.798	0.041	65.492	2.562
	10	0.071	1283.244	0.052	73.51	17.457
	100	0.07	12762.767	0.053	79.056	161.440
m = 15,n = 20	1	0.206	174.048	0.086	61.276	2.840
	10	0.187	1404.385	0.131	72.257	19.436
	100	0.176	15215.566	0.12	70.671	215.301

**Table 2:** Simulation result for the perfect test cases (N = 100, L = 5).

System Scale	*mc*	Algorithm 1		Algorithm 2		*Ratio*
		Time(s)	Cost	Time(s)	Cost	
m = 10, n = 15	1	0.121	173.714	0.06	61.878	2.807
	10	0.117	1604.094	0.076	76.661	20.925
	100	0.113	14553.069	0.077	77.585	187.576
m = 15, n = 20	1	0.362	201.794	0.133	54.235	3.721
	10	0.381	1859.124	0.199	76.775	24.215
	100	0.345	17263.422	0.199	67.607	255.350

**Table 3:** Simulation result for the perfect test cases (N = 10, L = 3).

System Scale	*mc*	Algorithm 1		Algorithm 2		*Ratio*
		Time(s)	Cost	Time(s)	Cost	
m = 10, n = 15	1	0.301	19.928	0.059	8.849	2.252
	10	0.365	121.748	0.06	10.674	11.406
	100	0.385	1242.526	0.057	10.81	114.942
m = 15, n = 20	1	7.842	18.091	0.137	9.116	1.985
	10	1.613	162.558	0.149	11.171	14.552
	100	1.904	1578.83	0.138	10.819	145.931

**Table 4:** Simulation result for the perfect test cases (N = 1000, L = 3).

System Scale	*mc*	Algorithm 1		Algorithm 2		*Ratio*
		Time(s)	Cost	Time(s)	Cost	
m = 10, n = 15	1	0.124	1724.087	0.042	655.273	2.631
	10	0.124	14149.781	0.043	762.456	18.558
	100	0.126	125411.935	0.054	701.254	178.840
m = 15, n = 20	1	0.307	1733.173	0.092	520.284	3.331
	10	0.307	17354.28	0.093	691.246	25.106
	100	0.292	159739.741	0.116	675.67	236.417

*● Imperfect Test Scenario*

When the tests are imperfect, the dependent relationships between the tests and the faults are denoted by a probability. In this scenario, the density of the D-matrix is set as 30%, which means that 30% of the elements denote fault detection probability and the others denote false alarm. In the simulation, they are generated randomly in [0.9,1] and [0,0.05], respectively. The maximum backtrack number is set to 3. The other parameters are the same as those presented in the perfect test scenario.

**Table 5:** Simulation result for the imperfect test cases (N = 100, L = 3).

System Scale	*mc*	Algorithm 1		Algorithm 2		*Ratio*
		Time(s)	Cost	Time(s)	Cost	
m = 10, n = 15	1	0.682	273.147	0.332	159.535	1.712
	10	0.718	1873.166	1.256	175.134	10.696
	100	0.77	19489.007	2.73	221.956	87.806
m = 15, n = 20	1	1.779	266.709	0.422	122.345	2.180
	10	1.694	2310.156	1.037	132.335	17.457
	100	1.676	18850.753	2.779	134.432	140.225

**Table 6:** Simulation result for the imperfect test cases (N = 100, L = 5).

System Scale	*mc*	Algorithm 1		Algorithm 2		*Ratio*
		Time(s)	Cost	Time(s)	Cost	
m = 10, n = 15	1	1.122	243.99	0.377	121.237	2.013
	10	1.552	2441.38	2.474	152.468	16.012
	100	1.564	21749.518	3.776	147.585	147.369
m = 15, n = 20	1	2.64	283.033	0.538	121.973	2.320
	10	3.522	2520.372	1.695	125.558	20.073
	100	4.286	22570.595	5.093	142.808	158.049

According to the result shown in Table 1, Table 2, Table 3, Table 4, Table 5 and Table 6, we can obtain the following insights and conclusions:

(1) From all the results shown in Table 1, Table 2, Table 3, Table 4, Table 5 and Table 6, we can see that Algorithm 2 is better than Algorithm 1, *i.e*., ratio>1. The computational time is acceptable.
(2) With the increase of mode transition cost *i.e.*, *mc*, the advantage of Algorithm 2 is more and more obvious. This is because the mode transition cost constitutes a larger part of the total cost of the diagnostic strategy when *mc* becomes larger. It is reasonable that the algorithm designed for multimode system generates a better result. For the similar reason, Algorithm 2 becomes much better than Algorithm 1 when *N* is larger, which can be seen by comparing Table 1, Table 3 and Table 4.
(3) From the comparison between Table 1 and Table 2, and Table 5 and Table 6, we can see that the number of system modes *L* influences both the computational time and the efficiency of the algorithms. When *L* becomes larger, the computational time becomes larger because the system is more complicated. Algorithm 2 is slightly better than Algorithm 1, because more mode transitions may occur in the diagnostic strategy and Algorithm 2 has the ability to choose the optimal mode in the generation process.
(4) The computational time is longer in the imperfect test cases than that in the perfect test cases. This is reasonable because the probability calculation in the imperfect test scenario is more complicated than Boolean calculation in the perfect test scenario. Furthermore, more tests are needed to isolate a fault when the tests are imperfect, which means that the number of nodes in the diagnostic tree is larger. And thus more calculation is needed to generate the diagnostic strategy. However, the result of Algorithm 2 is still better than Algorithm 1 in the imperfect scenario.

In general, Algorithm 2 is better than Algorithm 1 in practical application. It has an acceptable computational efficiency and a result with lower cost, and can be used for both perfect test cases and imperfect test cases. 

## 6. A Real World Case

In this section, we use a combinational circuit taken from [17] as an example to evaluate the effectiveness of the algorithms proposed in this paper. This system consists of 10 faults, 13 tests and two system modes, which means that m = 10, n = 13, *L* = 2. *N* is estimated as *N* = 1000. The D-matrix is shown in Table 7.

**Table 7:** D-matrix of the real world system.

Mode 1										Mode 2					
	t1	t2	t3	t4	t5	t6	t7	t8	t9–t13	t1–t8	t9	t10	t11	t12	t13
f1	0	0	0	1	0	0	1	0	0	0	0	0	0	0	0
f2	0	1	0	0	1	0	0	0	0	0	0	0	0	0	0
f3	0	0	0	1	0	1	0	0	0	0	0	0	0	0	0
f4	1	1	1	1	1	0	1	0	0	0	1	1	0	1	0
f5	1	1	1	1	1	1	0	0	0	0	0	1	1	1	0
f6	0	0	0	0	0	1	0	1	0	0	0	0	0	0	0
f7	0	0	0	0	0	1	1	1	0	0	0	0	0	0	0
f8	0	0	0	0	0	0	1	1	0	0	0	0	0	0	0
f9	0	0	0	0	0	1	0	1	0	0	0	0	1	0	1
f10	0	0	0	0	0	0	1	1	0	0	1	0	0	0	1
f11	0	0	0	0	0	0	0	0	0	0	0	0	0	0	0

The probability of system OK is 0.9, *i.e.*, $p_{11}=0.9$. The others are 0.01. By analyzing the system, we set the mode transition cost as:

(19) $$C=\left[\begin{array}{cc} 0 & 0.05\\ 0.05 & 0 \end{array}\right]$$

Considering the positions of the tests, their costs are set as shown in Table 8.

**Table 8:** Test cost of the case.

Test Name	t1	t2	t3	t4	t5	t6	t7	t8	t9	t10	t11	t12	t13
CP	0.5	0.5	0.5	0.5	0.5	0.5	0.5	0.5	1	1	1	1	1
CE	0.05	0.05	0.05	0.05	0.05	0.05	0.05	0.05	0.03	0.03	0.03	0.03	0.03

**Figure 2:**
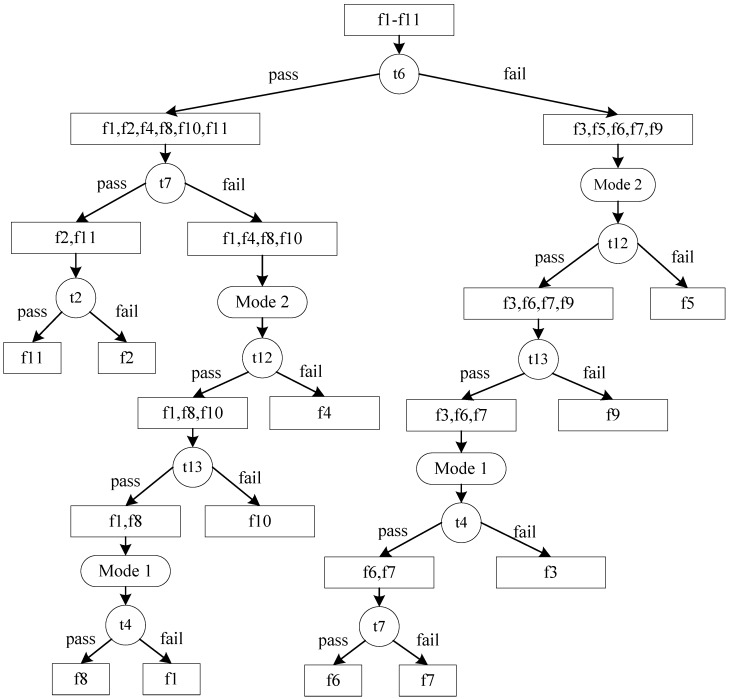
Diagnostic strategy generated by Algorithm 1.

**Figure 3:**
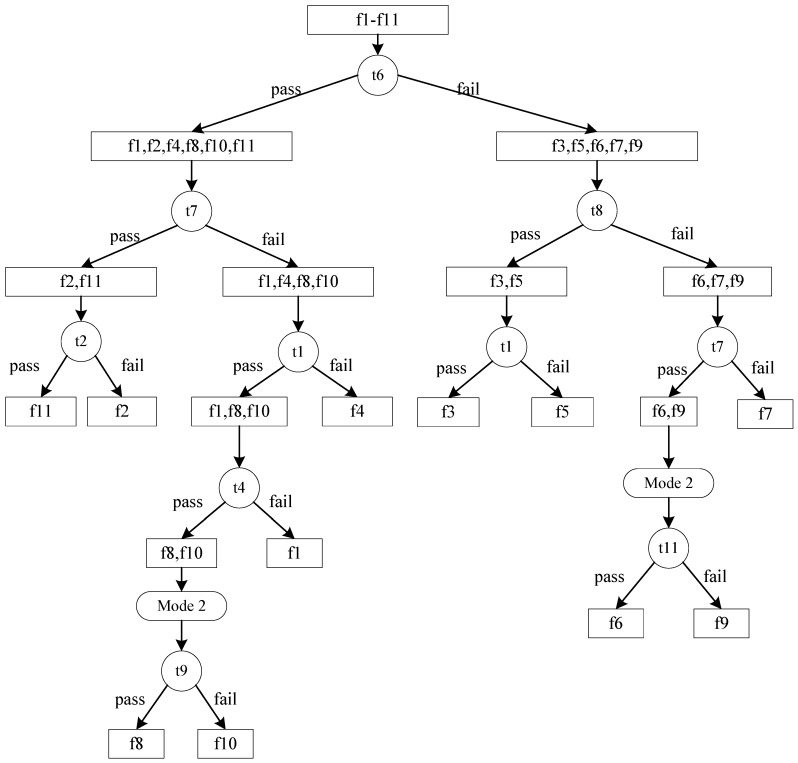
Diagnostic strategy generated by Algorithm 2.

Two different diagnostic strategies are generated using the algorithms proposed in this paper. The results are shown in Figure 2 and Figure 3. For Algorithm 1, the calculation time is 0.110 s, test cost of the diagnostic strategy is 164.421. The corresponding parameters of Algorithm 2 are 0.059 and 159.709, respectively. From the result, we can see that Algorithm 2 has a better result, which is consistent with the simulation results and our previous conclusions.

## 7. Conclusions

The optimal sequential diagnostic strategy generation problem considering test placement cost for multimode systems is studied in this paper. It is formulated as an AND/OR graph search problem. Two algorithms are proposed to solve the problem. One is realized by system transformation and the other is newly developed. Simulations are carried out to test the algorithms. The cases with different number of modes, mode transition cost, size and so on are studied. The result showed that both of them can solve the diagnostic strategy generation problem. Algorithm 2 is much better than Algorithm 1, and it is recommended to be used in the practical application. The algorithms are applied to a real-world case. The result is in agreement with the simulation data.

In the future, the diagnostic strategy generation algorithm for the multimode system with hierarchical structure will be developed. Then, the algorithm can be used to generate diagnostic strategies for the maintenance engineers of different level. Furthermore, test delay, multiple faults, fault propagation and other complicate scenarios can be studied.
